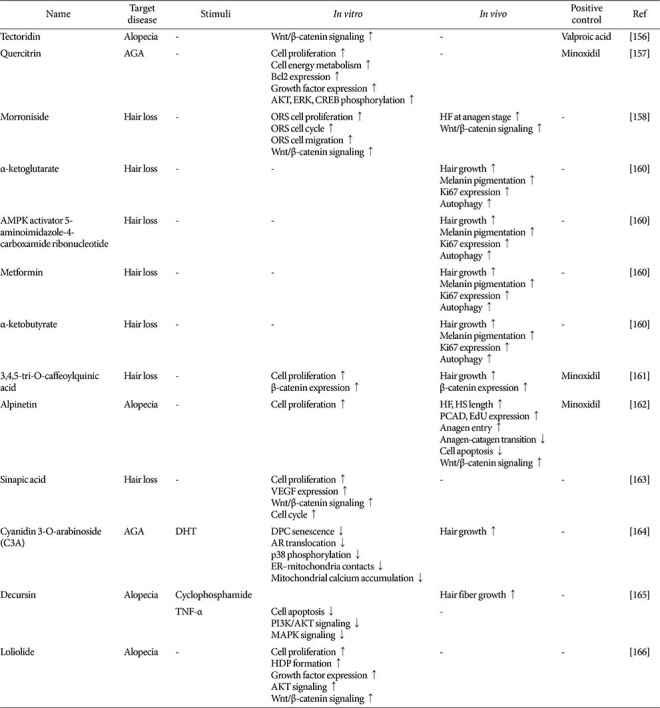# Phytochemicals for Hair Health Targeting Growth Signaling Molecules in Hair Follicular Stem Cells: A New Strategy for Hair Growth

**DOI:** 10.4014/jmb.2508.08030

**Published:** 2025-10-28

**Authors:** So Jeong Paik, Ming Zhang, Ha Yeong Kang, Min Jeong Woo, Hee Jung Choi, Sooah Kim, Sung Keun Jung

**Affiliations:** 1 School of Food Science and Biotechnology, Kyungpook National University, Daegu 41566, Republic of Korea; 2 Department of Environment Science & Biotechnology, Jeonju University, Jeonju 55069, Republic of Korea; 3 Research Institute of Tailored Food Technology, Kyungpook National University, Daegu, Republic of Korea

**Keywords:** Hair, hair follicle, pathways, phytochemicals, hair growth

## Abstract

Hair loss is a global concern, driving substantial public interest in effective therapeutic solutions. Beyond its protective physiological functions, hair plays a pivotal role in social and non-verbal communication, which can be profoundly disrupted by hair loss. While conventional pharmacological treatments are commonly employed, their use is often limited by the risk of adverse effects. To address this limitation, we propose the exploration of phytochemicals derived from natural sources as safer and potentially effective alternatives for hair loss prevention. Many natural compounds have shown the capacity to activate key signaling pathways involved in hair growth, offering promising avenues for innovation in the hair industry. This study explores the biological mechanisms underlying hair development, morphogenesis, and regeneration, with a particular focus on the Wnt/β-catenin, Sonic hedgehog (Shh), and Janus kinase (JAK)-signal transducer and activator of transcription (STAT) signaling pathways, which are critical to hair growth. Furthermore, we provide a comprehensive compilation of natural materials known to promote hair growth and their associated phytochemicals. This repository serves as a foundation for identifying and developing novel agents to enhance hair regeneration. Our review highlights the need for continued research into identifying and refining safe, naturally derived candidates for the prevention and treatment of hair loss, thereby addressing a pressing unmet medical and cosmetic need.

## Introduction

Mammals exhibit traits that differentiate them from non-mammals, with hair being one of the most distinctive. In humans, hair growth occurs on all skin surfaces except palmoplantar regions, such as the palms and soles [1], and hair plays an essential role in homeostasis by providing protection against external damage and contributing to thermoregulation. For example, scalp hair shields the head and neck, while eyelashes and eyebrows prevent contaminants from entering the eyes [2]. Hair also serves sensory functions, enhancing the skin’s ability to detect sensory stimuli [3]. Moreover, hair serves as a medium for social communication, and accordingly, disorders affecting hair can lead to considerable psychological distress, manifesting as depression, diminished confidence, and anxiety. Notably, personality disorders are more prevalent among men experiencing hair loss than among women experiencing hair loss [4].

The condition of hair is influenced by multiple factors, including sex steroid hormones, stress, ultraviolet (UV) exposure, smoking, microbial infections, environmental pollutants, irritants, and chemical exposure. Among these, sex hormones, particularly androgens, play a central role in androgenetic alopecia (AA), a common form of hair loss [5]. Additionally, exogenous factors that induce oxidative stress have deleterious effects on hair growth and retention [6]. Consequently, mitigating these detrimental factors is vital for maintaining hair health.

Currently, a range of hair growth treatments, including oral medications, topical applications, platelet-rich plasma therapy, laser therapy, and microneedling, is available [7]. Conventional hair growth therapies frequently target the regulation of sex hormones, but these are limited by significant side effects [8]. This underscores an urgent need for the development of safe and effective alternatives that minimize side effects. Botanical products have emerged as promising alternatives to synthetic treatments for preventing hair loss and promoting hair growth. Many plants contain diverse phytochemicals that synergistically enhance hair health [9]. Importantly, phytochemicals derived from botanical sources generally exhibit fewer and less acute toxic effects than synthetic drugs.

This review focuses on phytochemicals targeting hair growth-related molecular pathways as an innovative strategy for promoting hair health. We outline hair development processes, identify candidate compounds that modulate specific signaling pathways, and suggest ways to minimize side effects. The natural product-derived compounds regulating these cascades offer a promising and safe approach to effective hair growth treatments.

## General Description of Hair Development

### The Role of Hair

Hair originates at the base of the hair follicle (HF), which serves as the anchoring structure connecting the hair to the skin. Hair performs physiological functions, helping to retain body heat, facilitate sweating, and disperse sebaceous secretions, as well as psychological functions [10]. Initial hairs, termed “lanugo,” develop during the fetal period. During childhood, hair is classified as “vellus,” whereas by adulthood, it has transitioned to “terminal” hair. Each stage exhibits distinct characteristics in terms of color, length, diameter, and hardness [11]. Hair growth occurs at an average rate of 10–15 mm per month. From childhood onwards, humans possess approximately 5 million hairs, with roughly 100,000 HFs located on the scalp [12].

### Composition and Morphology of the HF

Hair follicles, highly conserved skin appendages, function as complex mini-organs with critical physiological roles primarily related to hair growth. Located within the dermis, HFs are among the few organs in the human body capable of regeneration throughout life [10, 13]. The terminal HF has a total length of 3,864 ± 605 μm and a diameter of 172 ± 70 μm [14].

**Fig. 1:**
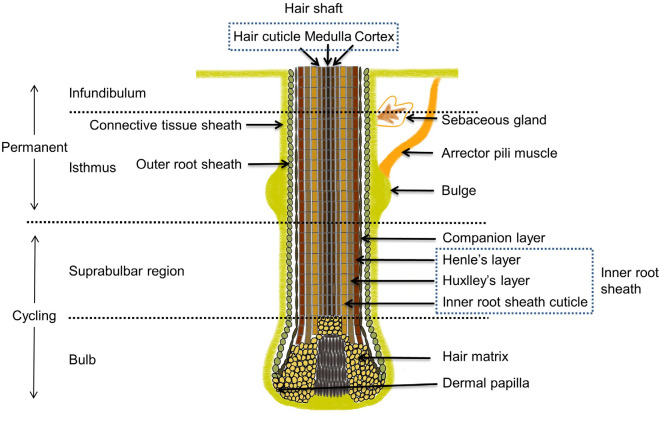
Histomorphology of the hair follicle.

The histomorphology of the HF is illustrated in Fig. 1, adapted from Schneider *et al*. [15]. Mature HFs are anatomically divided into two main regions: the upper and lower regions. The upper region comprises the infundibulum and isthmus, while the lower region includes the suprabulbar area and the bulb. The bulge, located within the permanent region, is a non-cycling zone. The permanent region also encompasses the arrector pili muscle and sebaceous glands, whereas the cycling region contains the dermal papilla (DP) and matrix. The DP, derived from mesodermal origin, influences the diameter and length of the hair shaft (HS), the size of the hair bulb (HB), and the duration of the growth phase [16, 17]. A longitudinal section of the HF reveals a cylindrical structure composed of eight distinct layers [18]. From the outermost to the innermost layer, the HF is organized into four major components: the inner root sheath (IRS), the outer root sheath (ORS), the associated layer, and the HS. The IRS is further subdivided into Henle’s layer, Huxley’s layer, and the IRS cuticle [19]. The HS itself consists of the medulla, cortex, and stratum corneum [20].

### HF Morphogenesis and Regeneration

Hair follicle morphogenesis and regeneration are typically considered as two interrelated phenomena [21]. Hair follicle morphogenesis predominantly occurs during embryonic development and involves intricate interactions between mesenchymal and epithelial tissues [22, 23]. While this process is primarily confined to the fetal stage, it has been demonstrated that new HFs can be induced postnatally through the transplantation of human HF dermal sheath tissue [24]. The progression of HF formation is delineated into eight specific stages, as outlined in Section HF Morphogenesis.

Upon the conclusion of HF morphogenesis, the follicle transitions into the regeneration phase, beginning with the formation of the HS. This phase is initiated by the shedding of the primary HS, which is subsequently replaced by a newly formed HS. Hair follicle regeneration commences during the catagen phase, which is marked by the postpartum shedding of lanugo hair and the emergence of vellus hair [25].

The prevailing consensus within the field is that HF regeneration proceeds through three cyclic stages: catagen (regression), telogen (quiescence), and anagen (growth) [11]. However, some researchers have proposed an alternative model incorporating a four-stage cycle, which includes the exogen phase, in which the existing mature HS is shed, as an independent and active process distinct from the telogen phase [26-28]. A key point of debate is whether the exogen phase is a passive component of telogen or represents a distinct, physiologically regulated stage. Milner *et al*. [28] provided evidence for the latter, demonstrating that hair shedding in mice involves active physiological regulation via protein hydrolysis. Further refinement of this concept was provided by Higgins *et al*.[23], who subdivided the exogen phase into “early exogen” and “late exogen.” Their analysis of mouse vibrissa follicles from the early anagen to late exogen stages revealed a progressive reduction in tissue attached to the club fiber, with minimal or no cellular material present in late exogen. These findings suggest that proteolysis facilitates the separation of the club fiber from the ORS, thereby enabling fiber shedding [29]. Moreover, experiments using desmoglein 3 gene-disrupted mice demonstrated that the absence of this gene leads to significant hair loss during telogen, underscoring its crucial role in anchoring hairs to the HF ORS [30]. Collectively, these findings support the notion that protein hydrolysis drives a transition from the telogen to a distinct exogen stage, reinforcing the concept of exogen as an autonomous and active stage of HF regeneration. Nevertheless, this interpretation remains a subject of ongoing debate.

Over the course of an individual’s lifetime, HFs undergo approximately 10–20 complete cycles, with scalp follicles cycling asynchronously [31]. Importantly, the hair growth cycle can be influenced by various exogenous factors, including hair plucking, skin trauma, temperature fluctuations, laser stimulation, and hormonal changes, which can accelerate or delay HF growth and influence hair vitality [32, 33].

## HF Morphogenesis

Hair follicle morphogenesis in mammals typically occurs once during the embryonic stage [34]. However, emerging evidence indicates that *de novo* HF formation can occur following wounding, as reported in several studies [35, 36]. Hair follicle formation is a dynamic process initiated by intricate signaling interactions between dermal mesenchymal and epithelial cells during embryogenesis [37]. It is widely accepted that the dermal mesenchyme functions as the inducer of HF formation, while the epithelium acts as the responder [38]. At approximately 50–60 days of gestation, epithelial cells respond to dermal signaling cues by forming the initial HF structure, referred to as the placode. These placode cells then transmit reciprocal signals to the underlying dermal mesenchyme, inducing dermal agglutination, a process primarily mediated by Wnt/β-catenin signaling pathways [37, 39].

Next, responding to Sonic hedgehog (Shh) signals from the dermal agglutination cluster, the epidermal placode invaginates into the dermis to form the primary hair germ. As growth progresses, the hair peg develops, culminating in the formation of the bulb, as aggregated dermal cells differentiate into the DP [39]. Signaling from the DP then stimulates rapid epithelial cell division, resulting in the encapsulation of the DP by HF epithelial cells and the formation of the HB. As the HF extends downward, the hair matrix (HM) undergoes rapid proliferation, migrates upward, and differentiates into the IRS and HS [40]. Concurrently, accessory HF structures, including the sebaceous glands and arrector pili muscles, are fully developed. At this stage, the differentiated HS begins to emerge from the scalp, with fully mature HFs producing the fine lanugo hair observed at birth [41].

**Fig. 2:**
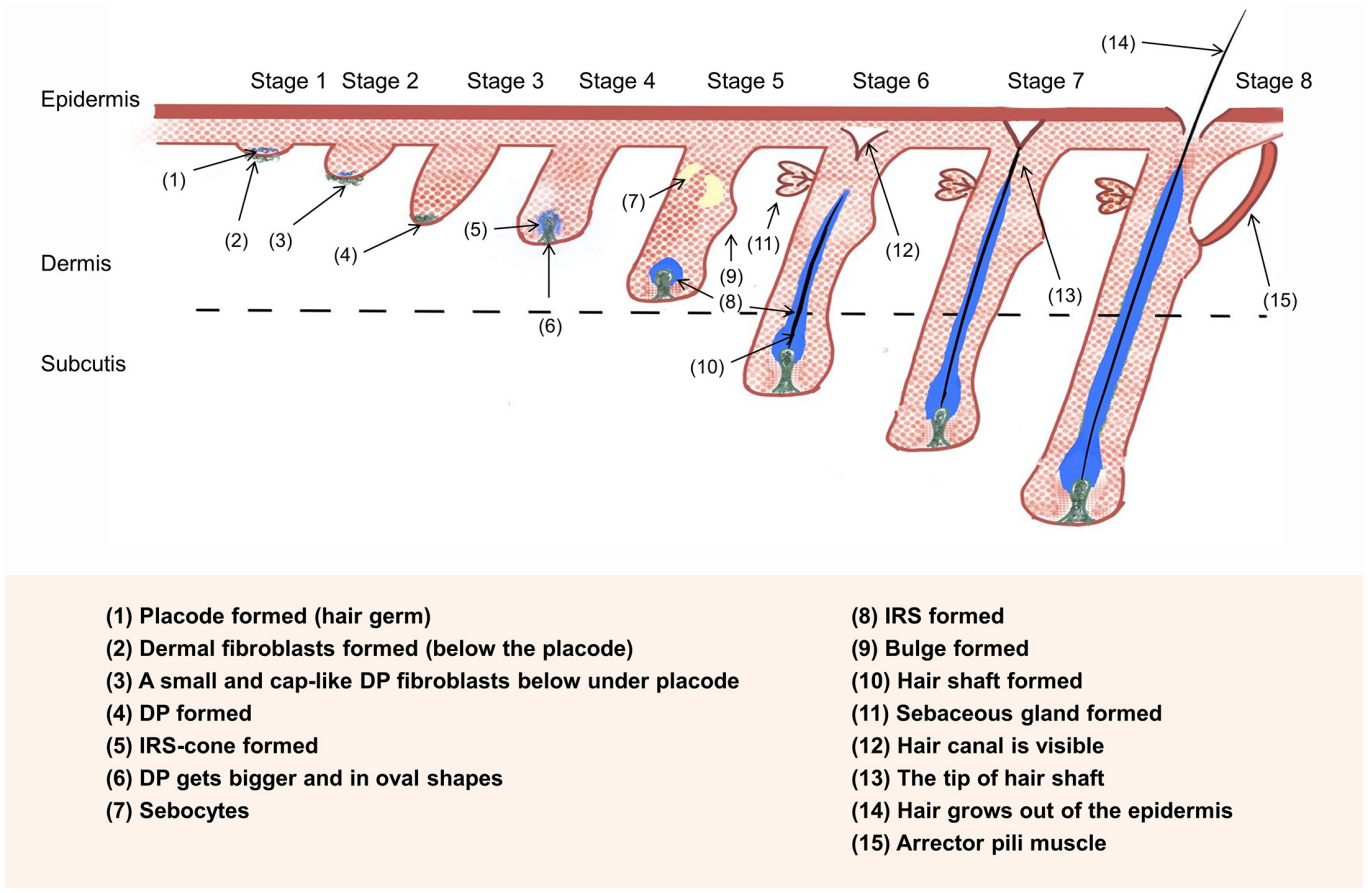
Overview of the hair follicle morphogenesis process.

Anatomical studies of rodent and human HFs have categorized HF development into eight distinct stages. Table 1 and Fig. 2, synthesized from multiple published studies and reviews, delineate the defining characteristics of each stage of HF morphogenesis [15, 25, 42-48].

## HF Regeneration

Hair follicles are cycling structures, undergoing continuous regenerative cycles throughout adult life, that serve as both stem cell reservoirs and HS factories [49-51]. While HF morphogenesis (embryonic organogenesis) and regeneration (postnatal regrowth) share several characteristics, they are distinct processes [21]. During regeneration, most HF structures arise from the inner matrix of the HB, with the ORS, derived from bulge cells, being the only exception [52]. In anagen, the ORS migrates downward as the IRS and HS ascend [53].

**Fig. 3:**
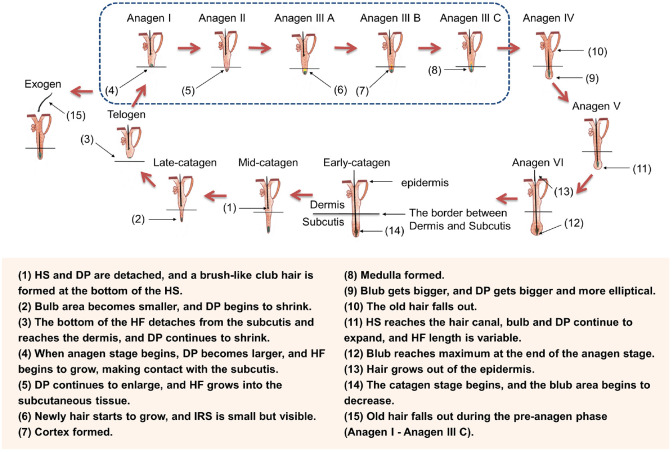
Overview of the hair follicle regeneration process.

Hair follicle regeneration is conventionally divided into three or four phases (Section 1.3), which can be further subdivided into multiple sub-phases. Using a murine model, Müller-Röver *et al*. [54] meticulously classified HFs at various stages, segmenting the anagen and catagen phases into eight sub-stages based on HF length and skin thickness. The HF’s length is shortest during telogen, increases to a maximum in anagen VI, remains stable until catagen II, and then shortens until the next telogen phase starts (Fig. 2) [54]. Each stage exhibits distinct characteristics, enabling accurate identification [25, 54, 55]. Table 2 summarizes these features, and Fig. 3 depicts them visually.

### Catagen: The Regression Phase

The catagen phase, the initial stage following HF morphogenesis, typically lasts approximately two weeks [47]. A defining feature of this phase is the formation of the club hair (CH), marking the separation of the HB from the DP [56]. Additional hallmarks of this phase include the cessation of terminal differentiation, apoptosis of keratinocytes, and halting of protein and melanin production within the regressing HB [57]. These prominent morphological changes distinctly signal the onset of catagen.

### Telogen: The Resting Phase

The identification of HFs in the telogen phase is relatively straightforward. In this stage, HFs are fully atrophied and located entirely within the dermis, with new HS formation not yet initiated [47]. Historically, telogen has been regarded as a resting phase; however, emerging evidence suggests ongoing physiological activity during this period. For example, expression levels of Cadherin 13 [58], Desmoglein 3 [30], and Keratin 24 [59] reach their peak during telogen.

### Exogen: The Active Shedding Phase

Traditionally, the mature HS is considered to be shed at the conclusion of the telogen phase or during the onset of the anagen phase (sub-stages anagen I–IV). However, as detailed above, some researchers classify this shedding process as a distinct phase termed “exogen,” aligning with the nomenclatural convention of the other phases [28, 51]. Recent studies have confirmed the presence of the exogen stage in the human scalp hair cycle. Unlike the telogen phase, in which hairs remain firmly anchored to the scalp, as they do in the anagen phase, during the exogen phase, hairs are easily removed from the HF without any attached cellular structures [23, 60].

### Anagen: The Growth Phase

The transition from telogen to anagen is marked by significant morphological and cellular changes. The pre-anagen phase closely resembles the late telogen, with a notable distinction being the enlargement and downward growth of the DP. Simultaneously, stem cells within the HF bulge rapidly proliferate, differentiating into new HS cells [25].

Throughout the anagen phase, HF keratinocytes exhibit vigorous proliferation, progressing through multiple sub-stages that culminate in the formation of the ORS, IRS, and HS. This phase typically spans 3–5 years, with hair growth occurring at an average rate of approximately 0.3 mm per day [61].

The regenerative capacity of HFs has been explored through several theoretical frameworks, including the epithelial theory, oscillating signal theory, bulge activation theory, papilla morphogen theory, resonance theory, inherent embryonic cycle theory, and inhibition-disinhibition theory [62]. However, the precise regulatory mechanisms underlying HF regeneration remain to be fully elucidated.

## Regulation of Hair Regeneration

Hair morphogenesis, generation, and growth are orchestrated by a complex interplay of multiple signaling pathways, including Hedgehog, Wnt, and bone morphogenetic proteins (Bmp2, Bmp4), which are essential for HF induction and morphogenesis [63]. These pathways interact synergistically to sustain the hair cycle, whereas their aberrant activation can cause hair dysplasia and skin cancer [64]. Despite considerable advances in this field, the precise mechanisms underlying HF formation and HF stem cell (HFSC) regulation remain incompletely understood.

### Wnt/β-catenin Signaling

The Wnt/β-catenin signaling pathway is initiated by the binding of Wnt proteins to cell membrane receptors [65]. In the absence of Wnt ligands, β-catenin is phosphorylated by glycogen synthase kinase-3β (GSK3β), axis inhibition protein (Axin), adenomatous polyposis coli (APC), and casein kinase 1 (CK1) within the destruction complex, leading to proteasomal degradation [66]. Afterwards, the Groucho/transducin-like enhancer of split (TLE) suppresses the lymphoid enhancer factor (LEF)/T cell factor (TCF)-mediated transcription of hair growth-related genes [67].

**Fig. 4:**
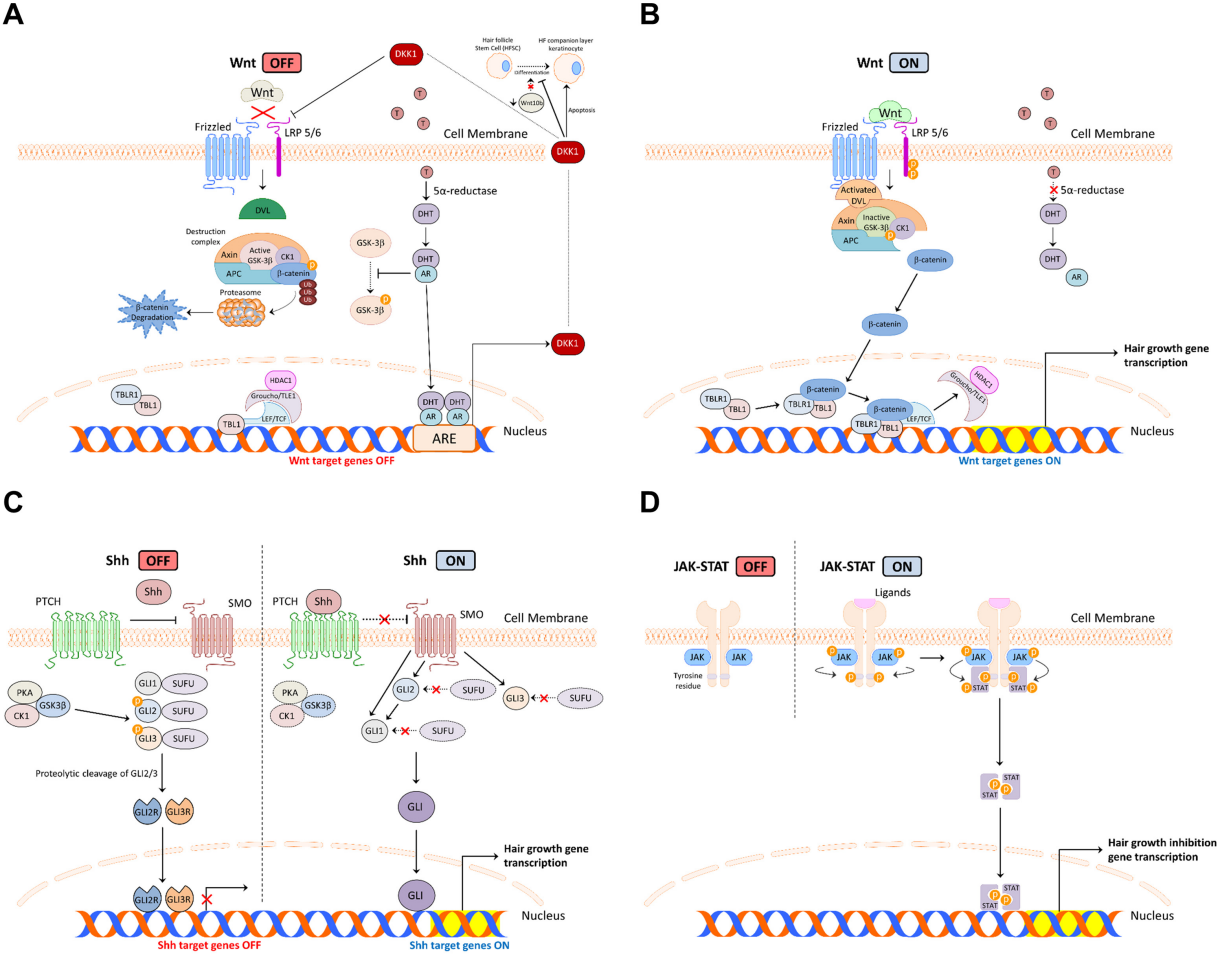
Key signaling pathways involved in the regulation of hair regeneration: the (**A**) inactivated and (**B**) activated states of the Wnt/β-catenin signaling pathway, (**C**) activated and inactivated states of the Shh signaling pathway, and (**D**) activated and inactivated states of the JAK-STAT signaling pathway.

Testosterone exerts a critical inhibitory effect on the Wnt/β-catenin signaling pathway. Upon entry into the cell, testosterone is converted to dihydrotestosterone (DHT) by 5α-reductase (5AR). Then, DHT binds to the androgen receptor (AR) and induces dickkopf-related protein 1 (DKK1) expression [68], suppressing HFSC differentiation and triggering catagen [69] (Fig. 4A).

Conversely, Wnt activation promotes the disheveled (DVL)-mediated inhibition of the dissemination of the destruction complex, enabling β-catenin to translocate to the nucleus and bind to transducin β-like protein 1 (TBL1) and TBL1-related protein (TBLR1). This complex, in turn, binds to LEF/TCF transcription factors, displacing Groucho/TLE1 and histone deacetylase 1 (HDAC1) to initiate target gene transcription [67, 70, 71]. Additionally, 5AR inhibition also prevents DHT-mediated DKK1 expression, sustaining hair growth [69] (Fig. 4B).

### Sonic Hedgehog Signaling in Hair Regeneration

The Shh signaling pathway is pivotal in HF development, with Shh being expressed in presumptive HFs [72]. In the absence of the Shh ligand, the 12-pass transmembrane receptor patched (PTCH) inhibits smoothened (SMO) activity [73]. When present, the Shh ligand binds to PTCH, relieving this inhibition and allowing the activation of SMO and the downstream glioma-associated oncogene homolog (GLI) family of transcription factors regulated by the suppressor of fused (SUFU) [74, 75]. By promoting the proteolytic cleavage of GLI2/3 into their repressor forms (GLI2/3R) that inhibit target gene transcription, SUFU functions as a negative regulator [76, 77]. Upon activation, GLI proteins translocate to the nucleus to induce the expression of genes (PTCH1, GLI1, cyclin D1, cyclin D2, and Sox9), crucial for HFSC maintenance [78, 79]. Experimental studies have shown that wild-type nude mice expressing the Shh gene continue to develop hair, whereas Shh mutants remain hairlessness and with pigmented skin [80]. The comprehensive Shh pathway is depicted in detail in Fig. 4C.

### JAK-STAT Signaling in Hair Regeneration

The JAK-STAT signaling pathway is implicated in numerous pathological conditions, including malignancies and autoimmune diseases, such as AA [81-84]. JAK kinases -JAK1, JAK2, JAK3, and tyrosine kinase 2 (TYK2)- phosphorylate STAT transcription factors upon cytokine- or growth factor-induced receptor dimerization [81, 85]. Phosphorylated STATs dissociate from the receptor, form homo- or heterodimers, and translocate to the nucleus [86]. In the nucleus, STAT dimers bind to specific DNA sequences to regulate gene expression, acting as transcriptional complexes. These transcriptional events ultimately inhibit hair growth [87], contributing to the pathogenesis of AA [84] (Fig. 4D). Thus, the modulation of JAK-STAT signaling represents a promising therapeutic strategy for addressing hair growth disorders.

## Mechanisms of Action and Adverse Effects of Hair Loss Management Drugs

Current pharmacological treatments for AA either promote hair growth or prevent hair loss, but their adverse effects necessitate a thorough understanding of their mechanisms of action and potential risks [88]. This section categorizes these drugs, summarizes their therapeutic efficacy, and outlines associated risks.

### Vasodilators

Vasodilators relax vascular smooth muscle, enhancing blood flow to regions with oxygen and nutrient deficiencies and supporting angiogenesis [89, 90]. Given the association between vasodilation and angiogenesis [91], promoting vascular dilation holds promise for stimulating hair growth. Topically administered minoxidil, a vasodilator-based treatment, has mechanisms of action that remain only partially elucidated. Minoxidil enhances the β-catenin signaling pathway in human dermal papilla cells (hDPCs), prolongs the anagen phase, and mitigates the transition to catagen in murine models, thereby promoting anagen phase maintenance [92]. It also downregulates AR-related functions [93], influences cell proliferation and apoptosis in hDPCs [94], and promotes root sheath outgrowth along the hair shaft in murine vibrissae follicle culture models [95]. Despite its well-documented efficacy, minoxidil is associated with adverse effects, including local erythema and pruritus, as well as other clinical symptoms [96-98].

### 5AR Inhibitors

As their name suggests, 5AR inhibitors specifically target 5AR, the enzyme responsible for the conversion of testosterone to DHT, a key androgen implicated in the pathogenesis of AA [99]. FDA-approved 5AR inhibitors effectively reduce the effects of DHT by inhibiting 5AR enzymatic activity [100].

Finasteride is an antiandrogenic agent that targets type II 5AR, facilitating hair regeneration by upregulating Wnt/β-catenin signaling through AKT phosphorylation and β-catenin activation. Additionally, finasteride enhances the expression of self-renewal transcription factors, such as Sox-2 and Nanog, in DP cells, promoting their aggregation and a stem cell-like phenotype [101, 102]. In animal studies, finasteride significantly reduced serum DHT levels and increased the number of anagen-phase follicles, which resulted in longer follicles and increased hair weights [103]. However, its use has been linked to severe adverse effects, including sexual dysfunction, orthostatic hypotension, and dizziness [104].

Unlike finasteride, dutasteride, another 5AR inhibitor, inhibits both type I and type II isoenzymes, resulting in a more pronounced suppression of serum DHT levels, as demonstrated in clinical studies [101]. Dutasteride effectively inhibited the formation of 5α-DHT, a testosterone metabolite, thereby mitigating AA [105]. Furthermore, dutasteride increases hair length and the proportion of HSs and HFs in the anagen phase [106]. Despite its therapeutic efficacy, dutasteride may cause sexual dysfunction (*e.g.*, decreased libido and impotence) and gynecomastia [88, 104].

### AR Antagonists

Dihydrotestosterone demonstrates a greater binding affinity for the ARs than testosterone, highlighting the critical role of ARs in androgen signaling pathways [107], and AR antagonists prevent androgens–AR interactions [108]. Additionally, nonsteroidal AR antagonists act through the competitive inhibition of AR binding, further impeding androgen signaling and mitigating hair loss [109].

The AR antagonist spironolactone reduces testosterone levels and blocks AR activity [110]. However, its clinical use is associated with gynecomastia, hyperkalemia, irregular menstruation, muscle cramps, hypotension, and decreased libido [111]. Another AR antagonist, cyproterone acetate, acts as a direct inhibitor of DHT–AR binding, while concurrently reducing the secretion of follicle-stimulating hormone and luteinizing hormone, leading to decreased testosterone levels [110]. It also significantly influences the proliferation of DP cells [112].

### Nonsteroidal AR Antagonist

Flutamide is a nonsteroidal AR antagonist that competitively inhibits the binding of DHT to ARs [113, 114]. Preclinical studies in murine models have demonstrated that flutamide treatment promotes hair regrowth and increases HS length and diameter [113, 115]. However, flutamide therapy is associated with libido reduction, gynecomastia, anxiety, rash, and somnolence [116].

### PGF_2_ Analogs

Prostaglandins (PGs) represent promising therapeutic targets for hair loss, with PGE_2_ and PGF_2α_ acting as potent hair growth stimulators in hair follicles [117]. Initially, PGF_2_ analogues were utilized for reducing intraocular pressure but were associated with hypertrichosis [118]. This phenomenon has since been leveraged to stimulate hair growth, first demonstrated by the application of PGF_2_ analogues to murine dorsal skin during both the telogen and anagen phases [119].

Latanoprost, originally approved as an ophthalmic solution [120], has been demonstrated to stimulate keratinocyte proliferation within HFs, thereby facilitating hair growth. Moreover, latanoprost enhances dermal vasodilation, improving HF nutrition through increased blood flow [121]. However, its use has been associated with alterations in eyelash morphology and the development of iridial and periocular hyperpigmentation [122]. A second, bimatoprost effectively increased both the number and length of HFs in the anagen phase in scalp follicle organ culture models and promoted hair regrowth *in vivo* [123], without notable adverse effects [124].

### Imidazole Antifungal Agents

Imidazole compounds, such as ketoconazole, well-known for their broad-spectrum antifungal activity, have also been shown to facilitate hair growth by inhibiting DHT production and/or binding to ARs [125, 126]. Ketoconazole upregulates OVOL1 expression at the cellular level, suggesting that its effects on hair growth may be mediated through the Wnt/β-catenin signaling cascade [127, 128]. Moreover, it has been shown to promote hair regrowth and increase HF diameter in male murine models [129]. Despite these therapeutic benefits, ketoconazole use is associated with decreased libido, nausea, vomiting, pruritus, and gynecomastia [103].

### JAK Inhibitors

JAK inhibitors have gained attention as targeted molecular therapies for AA, and the U.S. FDA has approved tofacitinib, ruxolitinib, and ritlecitinib for human use [130, 131]. Tofacitinib, a pan-JAK inhibitor, targets JAK1, JAK2, JAK3, and TYK2, while ruxolitinib specifically inhibits JAK1 and JAK2. Tofacitinib promotes hair follicle dermal papilla cell (HFDPC) growth, upregulates β-catenin and Gli1 expression, and regulates cell cycle progression [132-134]. On the other hand, ruxolitinib upregulates both the Wnt/β-catenin and JAK-STAT pathways. To date, these agents have not been associated with significant adverse effects [130]. Additionally, orally administered baricitinib, a JAK1/2 inhibitor, demonstrated effectiveness in stimulating hair growth in both patients with AA and murine models [135]. Although generally well-tolerated, baricitinib has been associated with transient adverse effects, including neutropenia and a reduced reticulocyte count, as reported in some studies [136]. Another orally administered JAK inhibitor, CTP-543, which has received a Fast Track designation from the FDA, has shown promise in promoting the anagen phase of HFs by inhibiting JAK1 and JAK2 [137]. Additional emerging agents, including ritlecitinib (PF-06651600) and brepocitinib (PF-06700841), have demonstrated the potential to facilitate hair regrowth through the inhibition of JAK activation [132, 137-139].

As previously discussed, numerous pharmacological agents carry the risk of serious systemic side effects. Consequently, ongoing research is focused on identifying drugs with a more favorable safety profiles, with a particular focus on those that promote hair growth via the Wnt/β-catenin or Shh signaling pathways [140] or by inhibiting the JAK-STAT pathway [132].

## Natural Nutraceuticals/Cosmeceuticals Affecting Hair Growth Signaling Pathways

Current pharmacological treatments for hair loss are effective but frequently associated with undesirable side effects, particularly sexual dysfunction. This has driven interest in safer alternatives that can promote hair growth without such complications. Offering a safer and potentially more sustainable approach, natural nutraceuticals and cosmeceuticals are increasingly recognized as promising candidates.

### Hair Growth-Promoting Natural Products and Their Mechanisms of Action

This section examines a range of natural extracts with demonstrated hair growth-promoting properties, underscoring their potential as safer alternatives to conventional treatments (Table 3).

The propolis derived from Philippines stingless bees has shown significant hair growth-promoting effects in murine HFs, increasing the number of HFs in the anagen phase with greater efficacy than minoxidil. Mechanistically, this propolis activated both the mRNA and protein expressions of key components within the Wnt/β-catenin signaling pathway, including Lef1, Wnt3a, β-catenin, and Bmp2 [141].

*Gardenia florida* fruit extract was shown to stimulate cell proliferation and upregulate Wnt/β-catenin signaling, vascular endothelial growth factor (VEGF), and TGF-β1 in hDPCs. In murine models, topical application of the extract to the dorsal skin induced hair regeneration and increased hair length [142].

*Mangifera indica* leaf extract downregulated DKK1 and SRD51A mRNA levels while upregulating the expression of Axin2, NKD1, and Myc. Furthermore, it reduced DKK1 mRNA expression in response to DHT. *In vivo*, a 1%topical formulation of the extract promoted hair growth and elongation in treated mice [143].

De-saponinated Camellia seed cake extract, a by-product of the *Camellia oleifera* seed oil extraction process, enhanced DP cell proliferation and facilitated entry into the anagen phase of the hair cycle. Additionally, it activated potassium channels, counteracting the inhibitory effects of tolbutamide (a potassium channel blocker) on DP cell proliferation. At the cellular level, the extract regulated cytokine expression and promoted the phosphorylation of ERK and AKT. Topical applications in a murine model induced hair growth and increased VEGF content in the skin [144].

Centipedegrass extract has been shown to significantly accelerate the hair cycle in hDPCs via activation of the β-catenin pathway. It also promoted cell proliferation, increased ALP expression, and upregulated hair cycle-related genes and β-catenin signaling molecules. In mice, this extract effectively transitioned hair follicles from the telogen to the anagen phase, outperforming minoxidil across all hair growth markers [145].

*Malva verticillata* extract exhibited cell proliferation activity, and linoleic acid, one of its bioactive components, activated the Wnt/β-catenin pathway, promoting hair regrowth in the presence of DHT [146]. Similarly, *Salvia plebeia* extract stimulated cell proliferation and upregulated HGF expression in hDPCs [147]. As epithelial cell growth is inhibited by androgen-inducible transforming growth factor-β1 (TGF-β1) [148], suppressing TGF-β1 expression offers a promising approach for promoting sustained hair growth. The *S. plebeia* extract not only inhibited TGF-β1 expression but also regulated the Wnt/β-catenin pathway and phosphorylated ERK and AKT in hDPCs. Additionally, in C57BL/6 mice, it facilitated hair growth and HF formation [147].

The titrated extract of *Centella asiatica* has been shown to promote hair growth in 3D spheroid cultures of human HFDPCs by increasing cell proliferation and spheroid diameter. While initial investigations suggested potential activation of Wnt/β-catenin signaling, subsequent findings indicated no effect on genes associated with this pathway. Instead, the extract appears to inhibit STAT activation [149].

The methanolic extract of the *Miscanthus sinensis* var. purpurascens flower significantly enhanced cell proliferation in hDPCs and activated ERK phosphorylation and β-catenin signaling. Topical application of the extract to telogenic C57BL/6 mice skin resulted in increased black skin pigmentation and extended HF length. Additionally, the extract suppressed the secretion of cytokines implicated in hair growth regulation, including TGF-β1 and VEGF [150].

The topical application of *Angelica sinensis* induced significant hair regrowth in depilated mice, evidenced by a transition of skin color from pink to grey, the emergence of new hairs, the transition of HFs from the catagen to anagen phase, and the restoration of HF sizes and HS lengths. Additionally, the treatment inhibited cell apoptosis by modulating the NF-κB and MAPK signaling pathways [151].

*Crataegus pinnatifida* extract was shown to promote hair growth in hDPCs through enhanced cell proliferation and activation of MAPK and AKT phosphorylation. Notably, oral administration of the extract facilitated hair growth and inhibited apoptosis in murine skin tissue more effectively than finasteride [152].

*Aconiti Ciliare Tuber* extract emerged as a promising botanical agent for hair growth following a screening of 800 natural products. This extract activated β-catenin target gene transcription factors, promoted neural progenitor cell differentiation, elevated ALP activity, and stimulated DP cell proliferation *in vitro*. It also induced early anagen phase initiation in a mouse model [153].

*Nephelium lappaceum* var. pallens (Hiern) Leenh. extract demonstrated preventive effects against AA in both *in vitro* and *in vivo* testosterone-induced hair loss models. The extract enhanced cell proliferation in the presence of testosterone in HFDPC cultures, and it accelerated hair growth, and upregulated PCNA and cyclin D1 expression in testosterone-induced hair loss model mice [154]. Similarly, *Terminalia bellirica* (Gaertn.) Roxb. fruit extract was found to counteract the testosterone-induced inhibition of hair growth in C57BL/6 mice. Oral administration restored hair growth markers, including cyclin D1 and PCNA, to near-pretreatment levels and activated Wnt/β-catenin signaling pathways. Furthermore, it increased HF density [155].

Overall, multiple natural products have demonstrated substantial hair growth-promoting effects. However, the clinical relevance of these products remains to be verified and their active compounds should be identified.

### Hair Growth-Promoting Phytochemicals and Their Mechanisms of Action

**Fig. 5:**
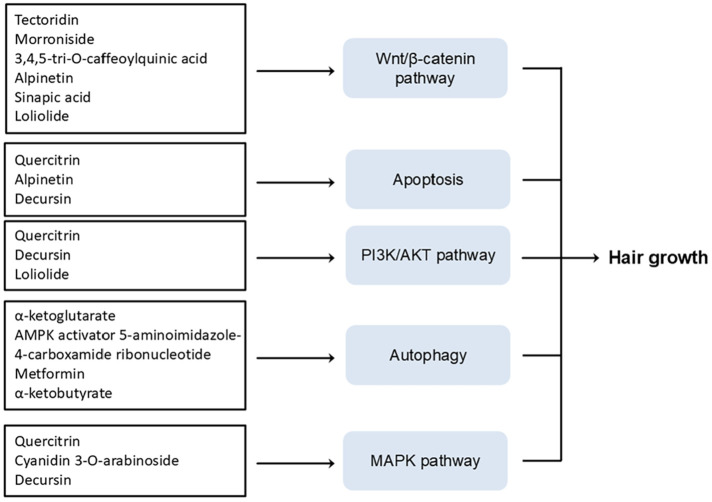
Known signaling pathways affecting hair health that are targeted by phytochemicals and pharmaceutical compounds.

Natural products are composed of a diverse array of components with intricate structures, and the specific bioactive compounds within a given extract determine its physiological effects. Identifying and characterizing compounds that promote hair growth is critical for developing novel therapeutic agents containing active ingredients. Here, we summarize key phytochemicals and their mechanisms of action, presenting a novel framework for advancing hair growth research (Table 4 and Fig. 5).

Tectoridin, an isoflavone derived from the rhizome of the Chinese medicinal herb *Belamcanda chinensis* (L.) DC., has been shown to activate the Wnt/β-catenin pathway by modulating luciferase activity and enhancing the expression of downstream target genes [156].

Quercitrin (quercetin-3-*O*-rhamnoside), a natural flavonoid found in various plants, predominantly in the flowers, fruits, and leaves, significantly enhanced cell proliferation in hDPCs, with effects comparable to those of 100 mM minoxidil. Furthermore, quercitrin increased mitochondrial membrane potential, a critical marker of energy metabolism. It also modulated the expression of apoptosis-related genes and proteins, upregulating Bcl2 and Ki67 while downregulating Bad and Bax. Additionally, quercitrin upregulated growth factor expression and phosphorylated AKT, ERK, and CREB-1, thereby activating both receptor tyrosine kinases and non-receptor tyrosine kinases [157].

Morroniside, a glycoside derived from *Cornus officinalis*, activates Wnt/β-catenin signaling, promoting ORSC proliferation and transition from the telogen to the anagen phase [158].

Autophagy plays a pivotal role in the differentiation and self-renewal of epidermal and dermal stem cells [159], and topical applications of α-ketoglutarate, the AMP-activated protein kinase activator 5-aminoimidazole-4-carboxamide ribonucleotide, metformin, and α-ketobutyrate enhanced hair regeneration by increasing melanin pigmentation, Ki67 expression, and autophagy-related protein expression in mouse skin tissue [160].

A polyphenolic compound 3,4,5-tri-O-caffeoylquinic acid stimulates hair growth via activation of the β-catenin pathway. Topical application of this compound prolonged hair length, an indicator of hair regrowth in mice. Microarray analysis revealed that skin treated with 3,4,5-tri-O-caffeoylquinic acid exhibited differential expression of 1,235 genes, including 435 upregulated and 800 downregulated genes. Of these, the upregulated genes were predominantly associated with the anagen phase and hair growth, while downregulated genes were linked to the telogen phase, apoptosis, cell cycle arrest, and repression of Wnt/β-catenin signaling. Enhanced β-catenin expression was observed in HFs and the epidermis of treated mouse skin. Furthermore, the treatment increased ATP content, ALP activity, and β-catenin expression in HFDPCs [161].

Alpinetin, a major constituent of herbs in the Zingiberaceae and Fabaceae families, has been shown to promote hair growth both *in vitro* and *in vivo*. In mice, alpinetin application altered skin pigmentation in mice, increased HF length 7 days post-depilation, and extended HS length at 13 and 17 days after depilation. It accelerated entry into the anagen phase and delayed the transition to the catagen phase by inhibiting cell apoptosis. Alpinetin also activated the expression of hair stem cell markers, and RNA-seq analysis revealed the upregulation of genes involved in cell migration, skin development, hair cycle regulation, and the Wnt signaling pathway. Importantly, alpinetin exhibited no cytotoxic effects in human and mouse primary fibroblasts and keratinocytes [162].

Sinapic acid, a hydroxycinnamic acid found in various herbs, vegetables, high-bran cereals, and fruits, has been proposed as a therapeutic candidate for hair loss. In HFDPCs, sinapic acid enhanced cell proliferation and increased VEGF production. Additionally, it promoted cell cycle progression by facilitating transitions to the S and G2/M phases, an effect mediated by AKT phosphorylation and the subsequent inactivation of the Wnt/β-catenin signaling [163].

Cyanidin 3-O-arabinoside protected DP cells from DHT-induced senescence and mitochondrial dysfunction by reducing mitochondrial ROS and regulating NADPH oxidase (NOX)-mediated MAPK signaling. It also suppressed the phosphorylation of p38 and HSP27 by regulating ROS formation in a NOX-dependent manner. Moreover, the compound inhibited ER–mitochondria contact and mitochondrial calcium accumulation. *In vivo*, the topical application of cyanidin 3-O-arabinoside accelerated hair growth in an AA-like mouse model [164].

Decursin, a pyranocoumarin compound and the primary bioactive component of *Angelica gigas*, was examined for its potential therapeutic effects on alopecia. A comprehensive literature review utilizing the PubChem database identified decursin as being closely associated with alopecia and implicated it in the apoptosis pathway related to chemotherapy-induced alopecia. In cyclophosphamide-treated mice, decursin reduced apoptosis markers and suppressed PI3K/AKT and MAPK signaling molecules [165].

Loliolide, a monoterpenoid, demonstrated therapeutic potential for alopecia by promoting the proliferation and 3D-culture formation of HDP cells. Additionally, the loliolide treatment activated the AKT signaling cascade, upregulating Wnt/β-catenin signaling via AKT pathway activation [166] (Fig. 5).

In this review, we systematically organized the known natural phytochemicals that exhibit promising effects on hair growth. Natural products contain a diverse array of bioactive compounds and, thus, exert multifaceted effects, necessitating the identification of key constituents in order to standardize product development for hair health. Despite extensive investigation into the efficacy of these compounds and the mechanisms driving their effects, none have yet translated into clinical applications. Consequently, further research is needed to evaluate phytochemicals with potential hair growth-promoting properties and minimal adverse effects on non-target organs.

## Conclusion and Future Research Strategies

Recent advancements in hair growth research have prioritized the development of safe and effective therapeutic materials with minimal adverse effects on non-target organs. A detailed understanding of the mechanisms regulating hair growth and the identification of treatment targets are essential for identifying new therapeutic candidates. In this review, we comprehensively examined the key aspects of the hair growth process, hair follicle morphogenesis and regeneration, the factors regulating follicle regeneration, current anti-hair loss drugs, and the novel hair growth-promoting agents.

Our review highlights the potential of natural product-derived phytochemicals as targeted modulators of molecular pathways involved in hair growth. Such approaches offer a promising avenue for maintaining hair health, accelerating the hair cycle, and promoting hair regeneration. Moreover, we revisited previously studied materials, offering innovative perspectives and suggesting strategies to refine and advance existing research paradigms in this field. These insights contribute to a more comprehensive understanding of the mechanisms promoting hair growth and support the development of effective phytochemical-based therapeutic agents that are both effective and exhibit minimal systemic side effects.

## Figures and Tables

**Table 1:** Key features of hair follicle morphogenesis.

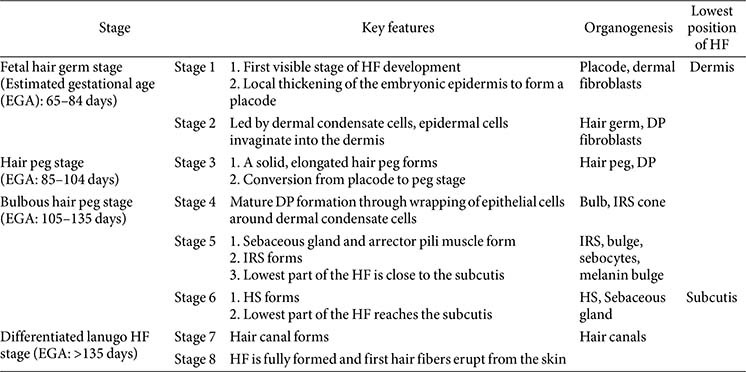

**Table 2:** Key features of hair follicle regeneration.

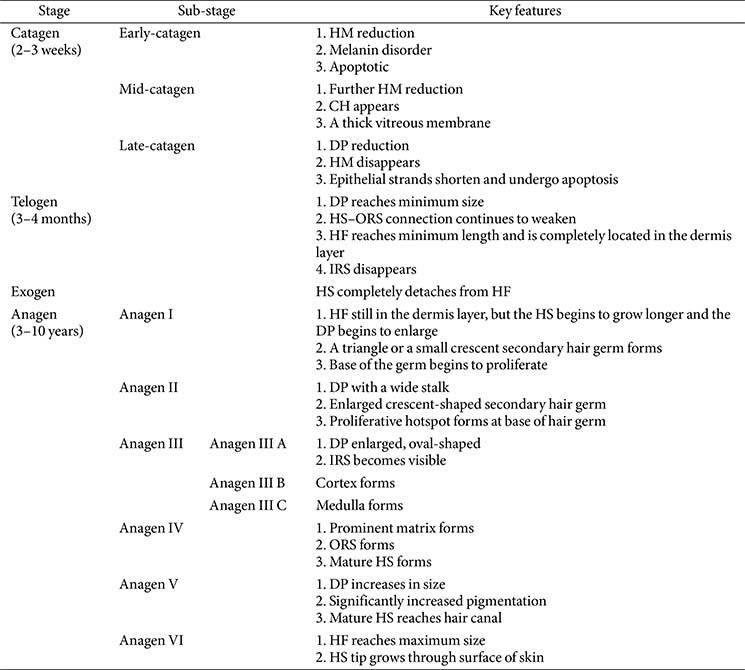

**Table 3:** Mechanisms of action of extracts promoting hair growth.

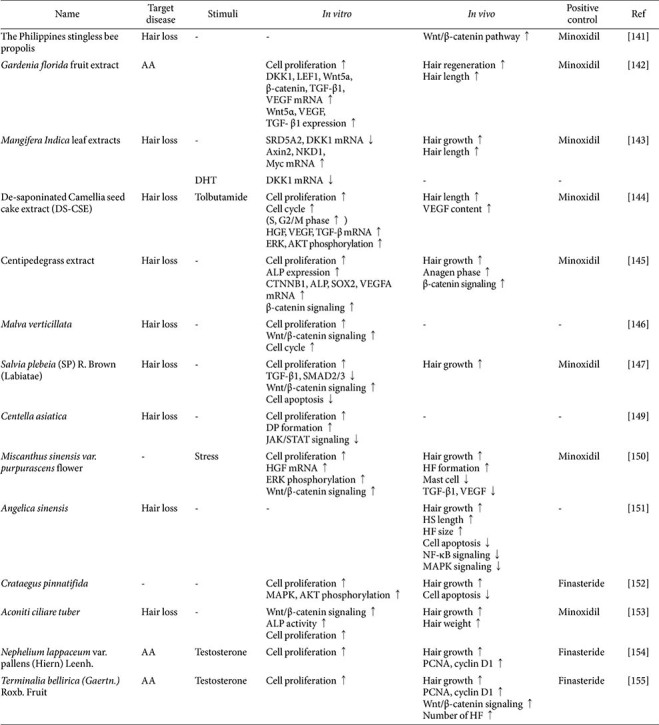

**Table 4:** Mechanisms of action of compounds promoting hair growth.